# Crosstalk between stromal components and tumor cells of TNBC via secreted factors enhances tumor growth and metastasis

**DOI:** 10.18632/oncotarget.19417

**Published:** 2017-07-21

**Authors:** Kideok Jin, Niranjan B. Pandey, Aleksander S. Popel

**Affiliations:** ^1^ Department of Biomedical Engineering, Johns Hopkins University School of Medicine, Baltimore, Maryland, USA; ^2^ Department of Oncology and Sidney Kimmel Comprehensive Cancer Center, Johns Hopkins University School of Medicine, Baltimore, Maryland, USA

**Keywords:** IL-8, TNBC, CXCR1/2, breast cancer, tumor microenvironment

## Abstract

Triple negative breast cancer (TNBC) as a metastatic disease is currently incurable. Reliable and reproducible methods for testing drugs against metastasis are not available. Stromal cells may play a critical role in tumor progression and metastasis. In this study, we determined that fibroblasts and macrophages secreted IL-8 upon induction by tumor cell-conditioned media (TCM) from MDA-MB-231 cancer cells. Our data showed that the proliferation of MDA-MB-231 cells co-cultured with fibroblasts or macrophages was enhanced compared to the monoculture. Furthermore, TNBC cell migration, a key step in tumor metastasis, was promoted by conditioned media (CM) from TCM-induced fibroblasts or macrophages. Knockdown of the IL-8 receptor CXCR2 by CRISPR-Cas9 reduces MDA-MB-231 cell proliferation and migration compared to wild type. In a mouse xenograft tumor model, the growth of MDA-MB-231-CXCR2^−/−^ tumor was significantly decreased compared to the growth of tumors from wild-type cells. In addition, the incidence of thoracic metastasis of MDA-MB-231-CXCR2^−/−^ tumors was reduced compared to wild type. We found that the auto- and paracrine loop exists between TNBC cells and stroma, which results in enhanced IL-8 secretion from the stromal components. Significantly, inhibition of the IL-8 signaling pathway by reparixin, an inhibitor of the IL-8 receptor, CXCR1/2, reduced MDA-MB-231 tumor growth and metastasis. Taken together, these findings implicate IL-8 signaling as a critical event in TNBC tumor growth and metastasis via crosstalk with stromal components.

## INTRODUCTION

Up to 10–15% of breast cancers do not express either estrogen receptor (ER)/progesterone receptor (PgR) or HER2 and are thus called triple-negative breast cancer (TNBC). TNBC can be particularly aggressive and is associated with poor outcome. Furthermore, TNBC is more likely to recur than other subtypes of breast cancer [1]. Chemotherapy is chosen for 90% of TNBC patients as a standard of systemic treatment due to lack of well-defined clinical targets of TNBC. Recently, however, several studies have focused on molecular targets such as PARP inhibitors, anti-androgen therapy, PI3K inhibitors, and MEK inhibitors to treat TNBC effectively [2]. Furthermore, many clinical trials have been testing immunotherapies such as the immune checkpoint inhibitors targeting PD-1/PD-L1 and CTLA-4 in TNBC patients [2]. The tumor microenvironment and its crucial emerging role in neoplastic diseases and metastasis are becoming widely recognized [3–9]. Cancer-associated fibroblasts (CAF) and tumor-associated macrophages (TAM) are major components of the tumor microenvironment, and they play critical roles in tumor initiation, maintenance, and progression. In addition, the tumor-associated stroma is involved in resistance to chemo-, targeted- and immuno-therapies [10–15], but the detailed mechanisms by which resistance develops are not well understood.

It is known that interleukin 8 (IL-8 or CXCL8) is secreted from leukocytes, fibroblasts, endothelial cells as well as tumor cells. IL-8 secreted from tumor cells is involved in angiogenesis, proliferation, and migration of tumor cells [16]. The IL-8 receptors, CXCR1 and CXCR2 form constitutive homo- and heterodimers selectively [17]. CXCR1 interacts with IL-6 and IL-8 while CXCR2 binds to IL-1, 2, 3, 5, 6, 7 and 8 [18]. IL-8 binds to CXCR2 with a higher affinity than to CXCR1 [19]. CXCR1 functions in IL-8 induced chemotaxis in immune response while CXCR2 plays a role in cell migration [20]. It has been reported that CXCR2 is a critical receptor for metastasis of several types of tumors [21, 22].

We have previously developed a robust metastatic model in which mice are pretreated with tumor cell-conditioned media (TCM) from human TNBC cells (MDA-MB-231 and SUM149) for two weeks prior to tumor cell inoculation. In this model, we found reproducible spontaneous metastases in lymph nodes (LN) and lungs within 4–5 weeks after orthotropic tumor inoculation [23]. We discovered that the TNBC tumor cells secrete large amounts of interleukin-6 (IL-6) that induced lymphatic endothelial cells (LEC) in the LN and lungs. Stat3, a transcription factor, gets activated by the IL-6 and induces the synthesis of CCL5 and VEGF among other factors from LEC. CCL5 recruits the tumor cells to the LN and lungs; VEGF helps build blood vessels in the LN to facilitate tumor cell survival; VEGF produced in the lung helps the tumor cells to extravasate into the lung. We have confirmed the importance of these factors by showing that inhibitors of these factors significantly inhibit metastasis.

Here, we show evidence for the function of IL-8 as a key secreted factor in TNBC tumor growth and metastasis through a crosstalk with fibroblasts and macrophages. Furthermore, we have identified CXCR1/2 as a potential therapeutic target in metastatic triple negative breast cancer.

## RESULTS

### Co-culture of TNBC cells with fibroblasts or macrophages promotes proliferation and migration

**Figure 1:**
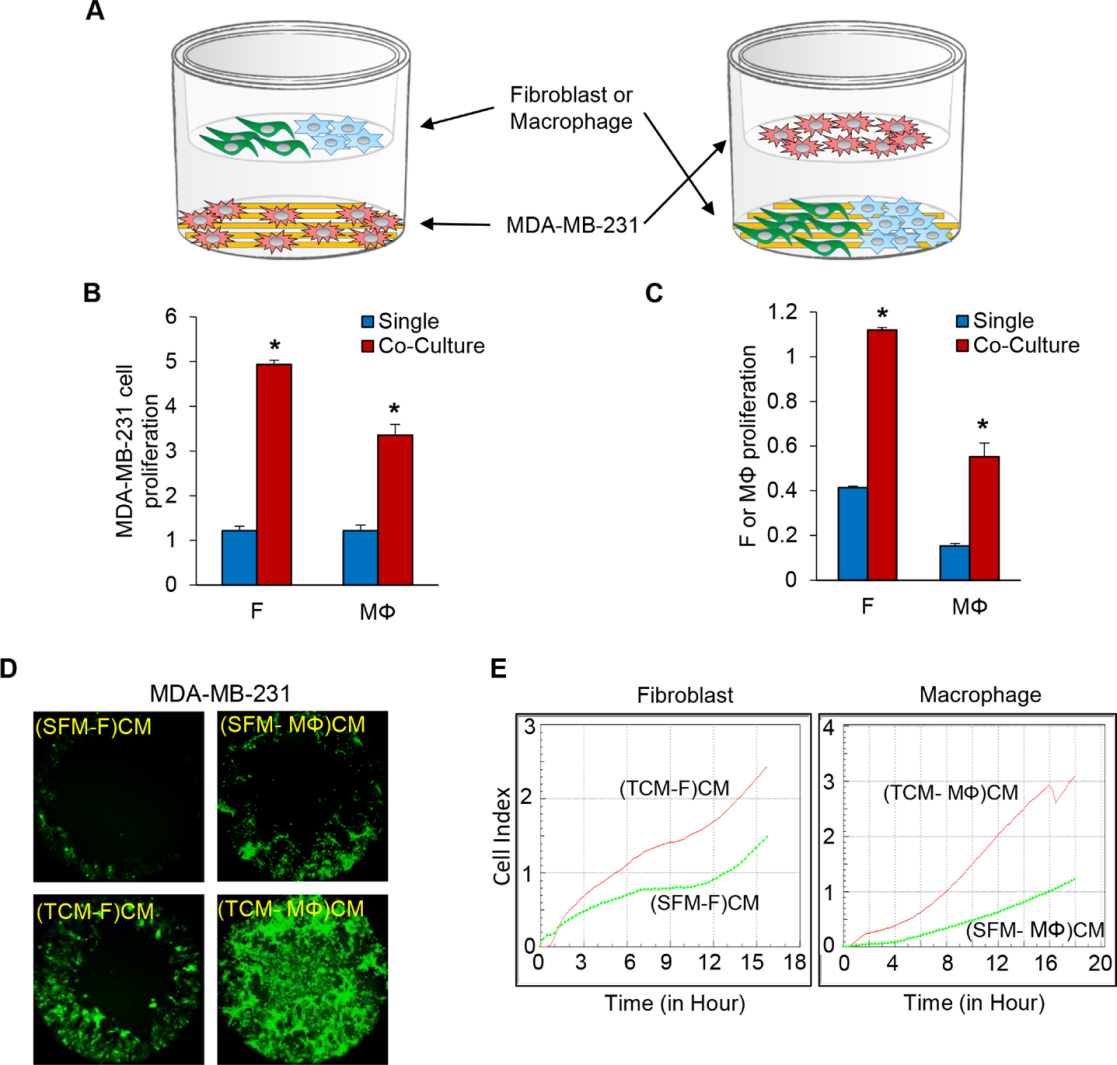
Co-culture of TNBC cells with fibroblasts or macrophages enhances cell proliferation and migration of the TNBC cells (**A**) Schematic diagram of MDA-MB-231 cells co-cultured with fibroblasts or macrophages (RTCA system, ACEA). (**B**) The proliferation of MDA-MB-231 cells in a co-culture with fibroblasts or macrophages. MDA-MB-231 cells were plated on the bottom chambers, and fibroblasts or macrophages (10,000 cells per well) were plated on the top chambers (E-plates insert). (**C**) The proliferation of fibroblast or macrophage cells in a co-culture of MDA-MB-231 cells. Fibroblasts or macrophages were plated on the bottom chambers, and MDA-MB-231 cells were plated on the top chamber. The bottom and top chambers were combined, loaded in the RTCA system and the cell index was measured continuously for 48 h (*P < 0.01, *n* = 3). (**D**) Migration of MDA-MB-231 cells pre-labelled with five uM Cell Tracker Green (CellTracker™ Green CMFDA, Thermo Fisher Scientific) for 30 minutes was assessed using the Oris cell migration kit (Platypus). Labeled MDA-MB-231 cells (50,000) in complete media were added to each well of a 96-well plate containing stoppers to prevent the cells from settling in the center region of the wells. The cells were allowed to adhere for 24 h, after which the stoppers were carefully removed. Conditioned media (CM) from fibroblasts or macrophages cultured with SFM (serum free media) containing with 2% serum or TCM (tumor conditioned media) of MDA-MB-231 cells were added, and the cells that migrated to the center of the well were observed after 48 h. CM was prepared by growing fibroblasts or macrophages in 30% SFM or TCM of MDA-MB-231 cells for four days after which the media were replaced with 3 ml SFM containing 2% FBS. After 48 h, the supernatant, also called the CM, was centrifuged and filtered. (**E**) Migration of MDA-MB-231 cells (top chamber) towards 180 ul of CM (bottom chamber) from fibroblasts or macrophages cultured with SFM containing 2% serum or TCM of MDA-MB-231 cells in the RTCA system. The cell index was measured continuously for 48 h. The migration profile of a representative experiment is shown. (SFM-F)CM and (SFM-MΦ)CM: conditioned media from fibroblasts (F) or macrophage (MΦ) cultured with SFM with 2% serum. (TCM-F)CM and (TCM-MΦ)CM: conditioned media from fibroblasts or macrophages cultured with TCM (tumor conditioned media) of MDA-MB-231cells. (*P < 0.01, *n* = 3).

It is well known that the crosstalk between cancer cells and stromal components in many tumor types can enhance tumor growth and metastasis [24, 25]. We have previously shown that the crosstalk between TNBC cells and LEC promotes tumor progression [23]. This finding motivated the hypothesis that TNBC cell proliferation and migration can be enhanced by co-culturing them with fibroblasts and macrophages. We co-cultured MDA-MB-231-luc-D3H2LN (referred to as MDA-MB-231 for brevity), SUM149, and SUM159 TNBC cells in E-plates (RTCA system from ACEA Biosciences, San Diego, CA) with normal fibroblasts and M2 type macrophage (Mϕ) differentiated from PBMC (Peripheral Blood Mononuclear Cells)/Monocytes in the co-culture insert. The same experiment was also done in the reverse orientation to investigate the effect of the cancer cells on the growth of the fibroblasts and macrophages. Co-culturing the different cell types enhanced the proliferation of both TNBC cells and fibroblasts or macrophages about 3 to 5-fold compared to the monocultures (Figure 1A–1C). In previous studies, we showed that the conditioned media of LEC induced by tumor conditioned media (TCM) of TNBC cells, enhanced migration of the TNBC cells [23]. We next investigated the effect of conditioned media of fibroblasts and macrophages induced by TCM of TNBC cells on the proliferation of MDA-MB-231 cells using E-plates (RTCA system ACEA) and on the migration of MDA-MB-231 cells using two different migration assays. We generated the conditioned media by culturing fibroblasts and macrophages with 30% TCM of MDA-MB-231 cells in growth media for three days and replacing to serum free media containing with 2% FBS. After 48 hours, the supernatant was collected and used for proliferation and migration assay.

Both proliferation and migration of MDA-MB-231 cells were significantly increased in the conditioned media of fibroblasts and macrophages induced by TCM of TNBC cells compared to conditioned media of fibroblasts and macrophages induced by serum free media (Figure 1D–1E and Supplementary Figure 1A–1E). These results suggest that the crosstalk between TNBC cells and fibroblasts or macrophages enhances migration and proliferation of the TNBC cells.

### TCM of MDA-MB-231 cells induces upregulation of IL-8 in fibroblasts or macrophages

**Figure 2:**
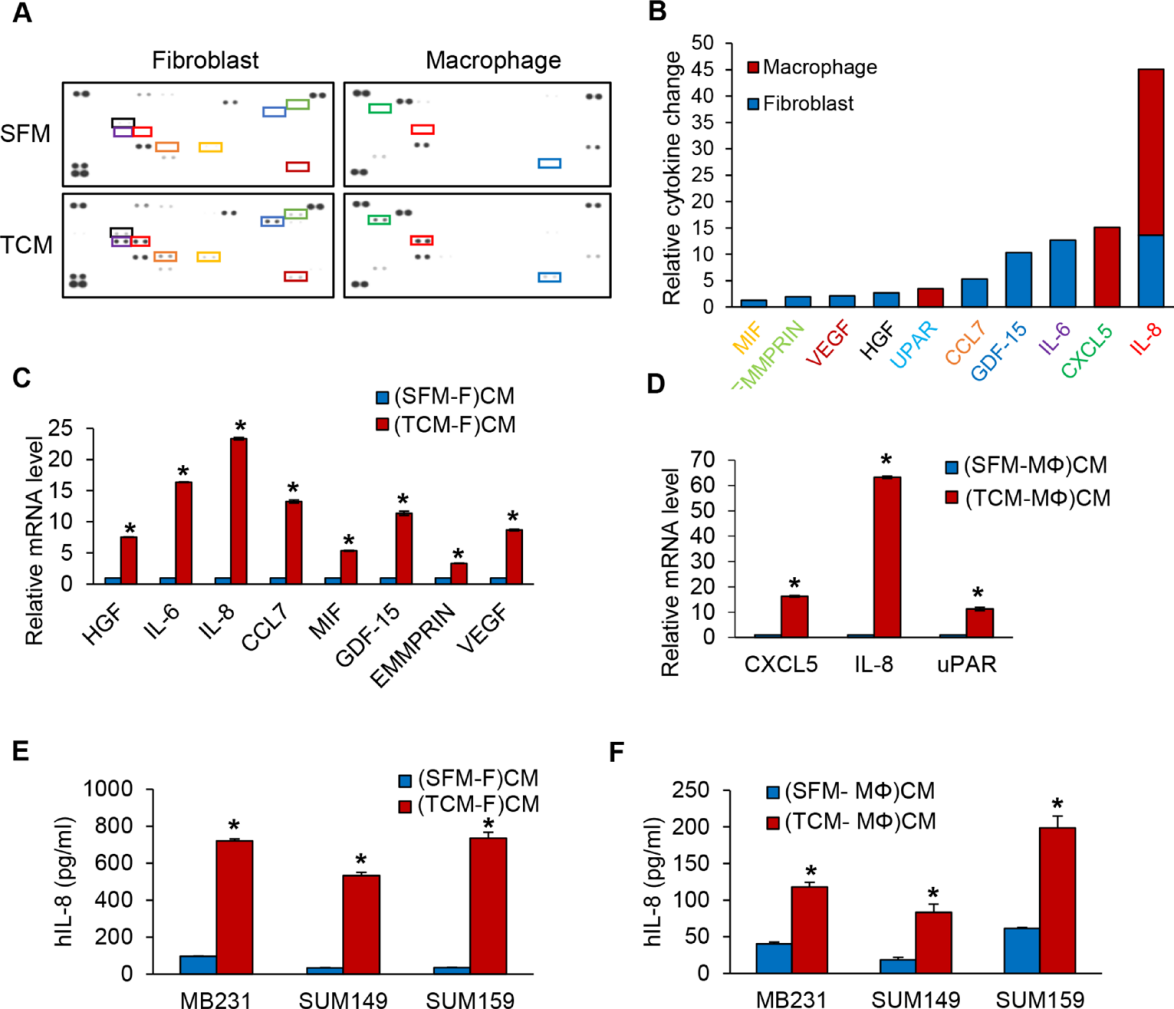
IL-8 protein expression is increased in fibroblasts or macrophages induced by TCM of TNBC cells (**A**) The relative amounts of cytokines present in CM of from fibroblasts or macrophages cultured with SFM containing with 2% serum or TCM of MDA-MB-231cells were visualized using a human cytokine antibody array (Proteome Profiler Human XL Cytokine Array Kit with 105 target proteins, R&D Systems). (**B**) A bar graph shows the relative changes in levels of the indicated cytokines. Profiles of mean spot pixel density were created by imaging analysis using ImageJ. (**C**) Real-time quantitative PCR analysis of cytokine mRNA levels in fibroblasts or (**D**) macrophages cultured with SFM containing with 2% serum or TCM of MDA-MB-231cells. (**E**) ELISA of human IL-8 (Quantikine ELISA, R&D System) in CM from fibroblasts or (**F**) macrophages cultured with SFM containing with 2% serum or TCM of TNBC cells. (*P < 0.001, *n* = 3). (SFM-F)CM and (SFM-MΦ)CM: conditioned media from fibroblasts (F) or macrophage (MΦ) cultured with SFM containing with 2% serum. (TCM-F)CM and (TCM-MΦ)CM: conditioned media from fibroblasts or macrophages cultured with TCM (tumor conditioned media) of MDA-MB-231cells.

In order to determine the secreted factors that are present in the conditioned media of fibroblasts induced by TCM of TNBC cells and in the conditioned media from macrophages induced by TCM of TNBC cells, could promote MDA-MB-231 cell proliferation and migration, we performed reverse western assays with a human cytokine antibody array (R&D Systems) targeting 105 cytokines. We discovered that HGF, IL-6, IL-8, CCL7, MIF, GDF-15, EMMPRIN, and VEGF were secreted by fibroblasts (fold change cut-offs of > 1.2) and CXCL5, IL-8, and uPAR were secreted by macrophages (fold change cut-offs of > 3.4) in response to induction by TNBC TCM (Figure 2A–2B). We selected IL-8 for further study because it was upregulated in both fibroblasts and macrophages. We confirmed that the expression and secretion of IL-8 was significantly increased from fibroblasts and macrophages induced by TCM of TNBC using real-time QRT-PCR and ELISA (Figure 2C–2F). These results suggest that IL-8 is highly secreted from fibroblasts and macrophages induced by TCM of TNBC cells and could be the factor that promotes the proliferation and migration of TNBC tumor cells.

### Decrease of TNBC cell proliferation and migration by inhibition of IL-8 signaling

**Figure 3:**
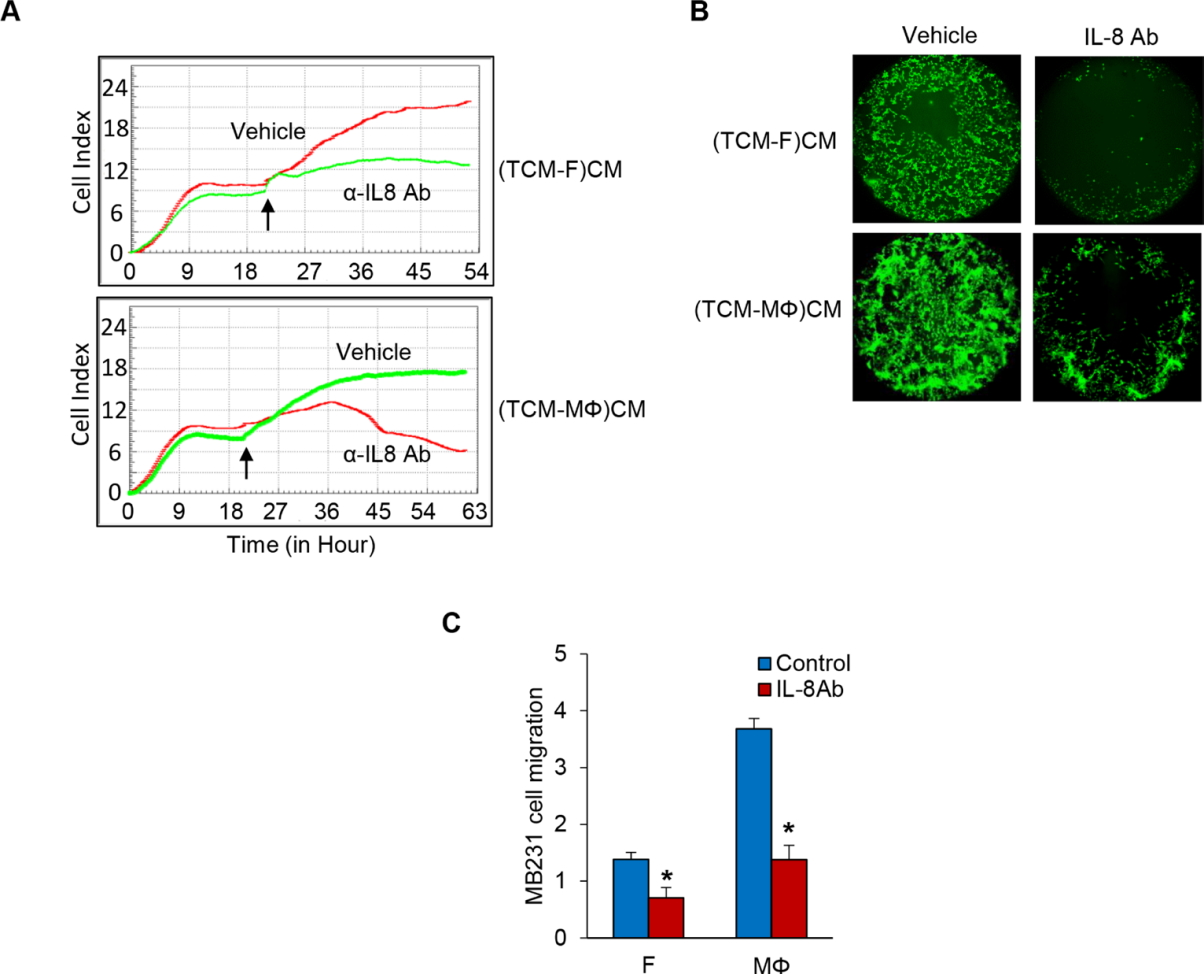
Inhibition of IL-8 blocks TNBC cell proliferation and migration (**A**) Proliferation assays of co-cultured MB231 cells on the bottom chambers with fibroblasts and macrophages treated with anti-IL-8 antibody (R&D Systems) in the top chamber in the RTCA system and the cell index was measured for 72 h. Arrows indicate the time of antibody adding top chamber. (**B**) Migration assay of MDA-MB-231 cells treated anti-IL-8 antibody in CM from fibroblasts or macrophages cultured with TCM of MDA-MB-231 cells using the Oris Cell Migration kit. (**C**) Migration assay of MDA-MB-231 cells (top chamber) treated anti-IL-8 antibody with CM (bottom chamber) from fibroblasts or macrophages cultured with SFM containing with 2% serum or TCM of MDA-MB-231cells in the RTCA system. The cell index was measured continuously at 48 h. The representative migration was shown. (*P < 0.01, *n* = 3). (TCM-F)CM and (TCM- MΦ) CM: conditioned media from fibroblasts or macrophages cultured with TCM (tumor conditioned media) of MDA-MB-231cells.

Next, in order to test if IL-8 is a key factor in the crosstalk between tumor cells and fibroblasts or macrophages in the regulation of TNBC cell proliferation and migration, we performed MDA-MB-231 cell proliferation and migration assays with conditioned media of fibroblasts or macrophage induced by TCM of TNBC cells in which the IL-8 is neutralized with an anti-IL-8 specific antibody. The results showed that neutralization of IL-8 significantly attenuated MDA-MB-231 cell proliferation co-cultured with fibroblasts and macrophages (Figure 3A and Supplementary Figure 2A). In addition, migration of TNBC cells was decreased about two fold in IL-8 neutralized conditioned media of fibroblasts or macrophages induced by TCM of TNBC cells compared to control (Figure 3B–3C and Supplementary Figure 2B–2F). These results show that IL-8 plays an important role in TNBC cell proliferation and migration.

### Knockout of CXCR2 using the CRISPR-Cas9 system inhibits proliferation and migration of MDA-MB-231 cells

**Figure 4:**
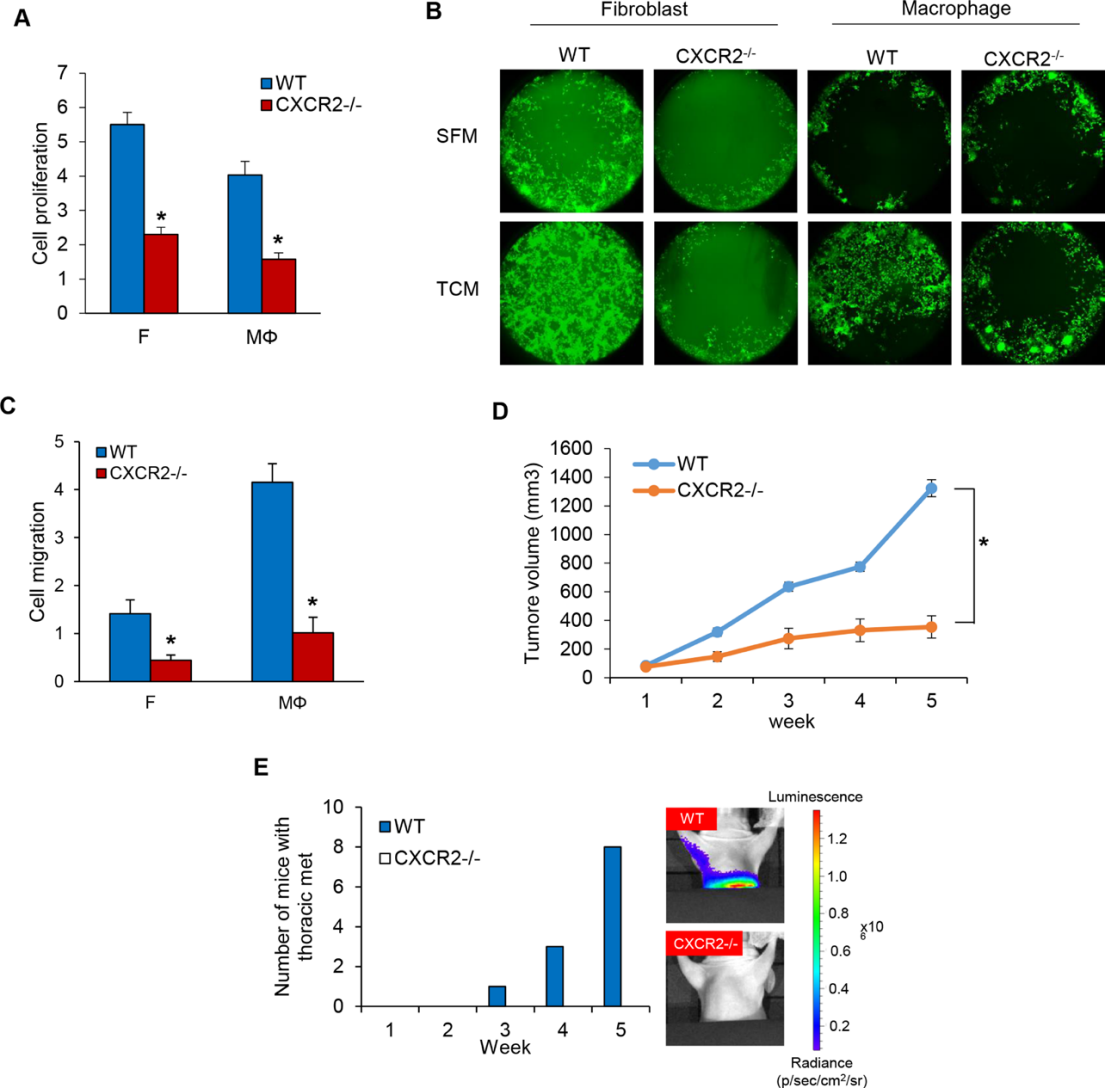
Knockout of CXCR2 using CRISPR-Cas9 system inhibits cell proliferation and migration in MDA-MB-231 cells (**A**) Proliferation assays of co-cultured MDA-MB-231-wild type and MDA-MB-231-CXCR2^−/−^ cells on the bottom chambers with fibroblasts and macrophages on the top chamber in the RTCA system (*P < 0.001, *n* = 3). (**B**) Migration assay of MDA-MB-231-wild type and MDA-MB-231-CXCR2^−/−^ cells in CM from fibroblasts or macrophages cultured with SFM containing with 2% serum or TCM of MDA-MB-231-wild type cells using the Oris cell migration kit. (**C**) Migration assay of wild type and CXCR2^−/−^ MDA-MB-231 cells (top chamber) with CM (bottom chamber) from fibroblasts or macrophages cultured with SFM containing with 2% serum or TCM of MDA-MB-231 cells using the RTCA system. The representative migration was shown (*P < 0.01, *n* = 3). (**D**) Tumor growth curves of MDA-MB-231-wild type and MDA-MB-231-CXCR2^−/−^ cells implanted mammary fat pad in athymic mice. (Mean ± SEM, *P < 0.001 and *n* = 8). (**E**) Athymic nude mice (4–5 weeks, female and Charles River) were pretreated with TCM (50 ul) of MB231 cells for two weeks before inoculation with MB231-WT and MB231-CXCR2^−/−^ cells. After five weeks, the number of mice with thoracic metastasis was counted using the IVIS imager.

Given the robust role of IL-8 in TNBC cell proliferation and migration via the crosstalk with fibroblasts and macrophages, we next tested the role of the IL-8 receptor (CXCR1/2) in TNBC cell proliferation and migration. We established a CXCR2 knockout MDA-MB-231 cell line using the CRISPR-Cas9 system (Supplementary Figure 3A) and assessed its proliferation and migration compared to that of wild type MDA-MB-231 cells. As predicted, both MDA-MB-231-CXCR2^−/−^ cell proliferation and migration were dramatically decreased compared to WILD TYPE (Figure 4A–4C and Supplementary Figure 3B and 3C). We next examined MDA-MB-231-CXCR2^−/−^ cell tumor growth and metastasis utilizing a model developed in our lab in which mice are pretreated with tumor cell-conditioned media (TCM) from human TNBC cells for two weeks prior to tumor cell inoculation to establish orthotopic tumor xenografts. The TNBC cells from these tumor xenografts metastasized robustly to lymph nodes (LN) and lungs so that in 4–5 weeks after tumor inoculation, 100% of the mice had metastases in these tissues [23]. MDA-MB-231-CXCR2^−/−^ cell tumor growth was significantly decreased compared to wild type. Furthermore, we observed no metastases in the lung and lymph nodes as shown by the reduced photon flux in these tissues. These results are in striking contrast with those of animals with orthotopic tumor xenografts generated from wild type MDA-MB-231 cells. The hearts, stomach, spleens, and livers did not show significant metastases. (Figure 4D and Supplementary Figure 3D and 3E). Using immunofluorescence and immunohistochemistry analysis of SMA, Iba-1, F4/80 and Ki-67, we found that both the proliferation and recruitment of fibroblasts and macrophages into the tumor was significantly decreased in MDA-MB-231-CXCR2^−/−^ cell tumors compared to tumors generated by wild type MDA-MB-231 cells (Supplementary Figure 3F–3L). The results suggest that the IL-8-CXCR2 axis plays a critical role in TNBC tumor growth and metastasis via the crosstalk of cancer cells with fibroblasts and macrophages.

### Paracrine IL-8 activates fibroblasts and macrophages

**Figure 5:**
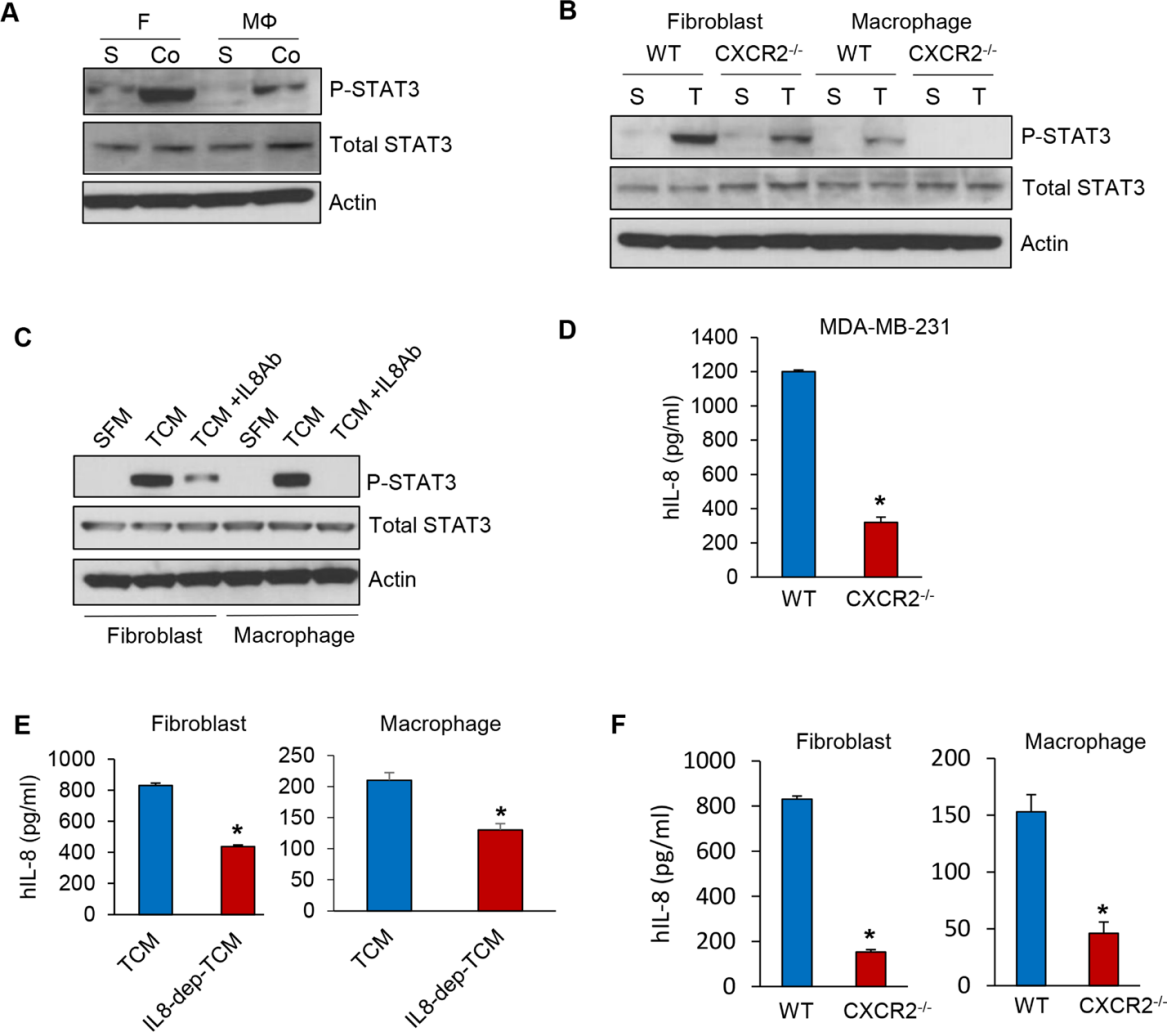
Auto- and paracrine loop of IL-8 exists between TNBC and stroma (**A**) Immunoblotting analysis of phosphorylated STAT3 in co-cultured MDA-MB-231 cells (Co) with fibroblasts and macrophages compared to single cultured MDA-MB-231 cells (S). (**B**) Immunoblotting analysis of phosphorylated STAT3 in MDA-MB-231-wild type and MDA-MB-231-CXCR2^−/−^ cells treated with CM from fibroblasts or macrophages cultured with SFM containing with 2% serum (S) or TCM (T) of MDA-MB-231-wild type cells. (**C**) Immunoblotting analysis of phosphorylated STAT3 in MDA-MB-231 cells treated with an anti-IL-8 antibody with the same condition as B. (**D**) ELISA of human IL-8 in TCM from the MDA-MB-231-wild type and MDA-MB-231-CXCR2^−/−^ cells (**E**) ELISA of human IL-8 in CM from fibroblasts or macrophages cultured with TCM of MDA-MB-231 cells treated anti-IL-8 antibody. (**F**) ELISA of human IL-8 in CM from fibroblasts or macrophages cultured with TCM of MDA-MB-231-wild type or MDA-MB-231-CXCR2^−/−^ cells. (*P < 0.01, *n* = 3).

Several studies have reported auto- and paracrine loops of IL-8 between the tumor cell and stromal components [26, 27]. Immunohistochemistry and immunofluorescence staining showed that the recruitment of cancer associated fibroblasts and tumor associated macrophages was significantly reduced in MDA-MB-231-CXCR2^−/−^ cell tumors (Supplementary Figure 3F and 3G). In addition, the migration of fibroblasts and macrophages was decreased in TCM of MDA-MB-231-CXCR2^−/−^ cells compared to TCM of wild type MDA-MB-231 cells (Supplementary Figure 3H). These findings led us to hypothesize that the paracrine IL-8 loop between TNBC cells and CAF or TAM regulates TNBC tumor growth and metastasis. To investigate the paracrine effect of IL-8 secreted by fibroblasts and macrophages on MDA-MB-231 cells, we first examined the activity of STAT3, a downstream effector of IL-8 signaling in MDA-MB-231 cells co-cultured with fibroblasts and macrophages. We found by immunoblotting that the level of phosphorylated STAT3 was significantly increased in MDA-MB-231 cells co-cultured with either fibroblasts or macrophages compared to MDA-MB-231 cells grown by themselves (Figure 5A). We next investigated the level of STAT3 phosphorylation in MDA-MB-231-wild type and MDA-MB-231-CXCR2^−/−^ cells treated with conditioned media of fibroblasts or macrophage induced by TCM of TNBC cells. Interestingly, we observed that the phospho-STAT3 levels were decreased in MDA-MB-231-CXCR2^−/−^ cells treated with conditioned media of fibroblasts or macrophage induced by TCM of TNBC cells compared to MDA-MB-231-wild type (Figure 5B). We next investigated if IL-8 secreted from MDA-MB-231 cells can activate fibroblasts and macrophages. We neutralized IL-8 protein in the TCM of MDA-MB-231 cells with an anti-IL-8 antibody and added the TCM to fibroblasts and macrophages. The phospho-STAT3 levels were dramatically decreased in fibroblasts and macrophages treated with IL-8 depleted TCM compared to TCM without treatment (Figure 5C). These results suggest that IL-8 from the crosstalk between MDA-MB-231 cells and fibroblasts or macrophages activates MDA-MB-231 tumor cells as well as the stromal fibroblasts and macrophages. To assess the relative levels of IL-8 secretion from the MDA-MB-231-wild type and MDA-MB-231-CXCR2^−/−^ cells, we performed ELISA analysis and found that the IL-8 levels were higher by about 3-fold in MDA-MB-231-wild type than in MDA-MB-231-CXCR2^−/−^ cells. This result suggests the lack of the autocrine IL-8 loop in MDA-MB-231-CXCR2^−/−^ cells (Figure 5D). Furthermore, we observed that IL-8 secreted by fibroblasts and macrophages cultured with IL-8 depleted TCM decreased about 2-fold in compared to that secreted by cells in control TCM (Figure 5E). In addition IL-8 was reduced about 2 to 3 fold in MDA-MB-231-CXCR2^−/−^ cells treated with conditioned media of fibroblasts or macrophage induced by TCM of TNBC cells compared to MDA-MB-231-wild type with the same conditioned media (Figure 5F). These data imply that activated auto- and paracrine loops of IL-8 from the crosstalk between TNBC cells and stroma play critical roles in TNBC proliferation and migration.

### Reparixin, a CXCR1/2 inhibitor, blocks TNBC cell proliferation and migration

**Figure 6:**
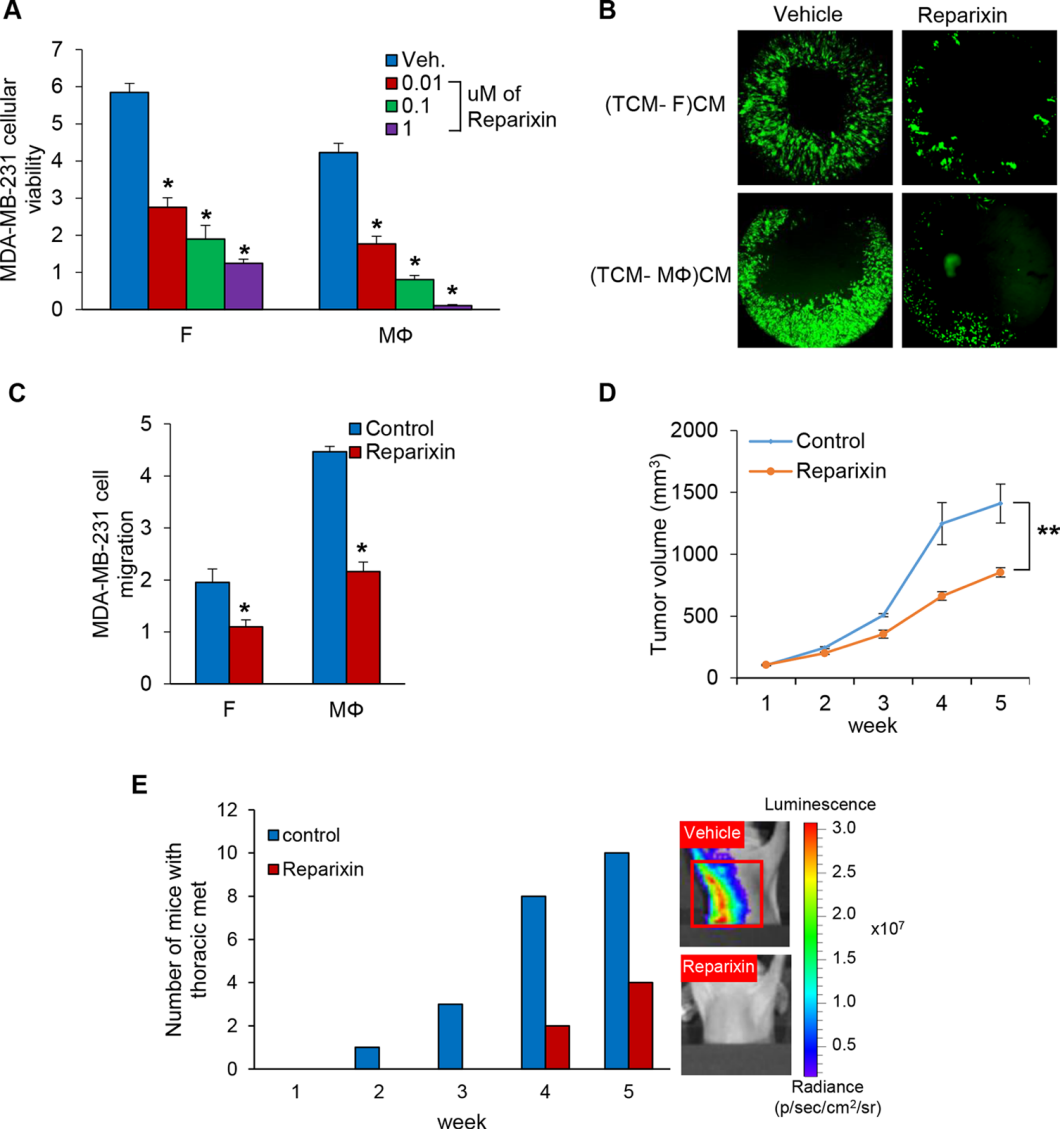
Reparixin, CXCR1/2 inhibitor blocks TNBC cell proliferation and migration (**A**) Cellular viability assays of co-cultured MDA-MB-231 cells on the bottom chambers with fibroblasts and macrophages treated 0.01 to 1 uM of reparixin on the top chamber in the RTCA system (*P < 0.001, *n* = 3). (**B**) Migration assay of MDA-MB-231 cells treated 0.1 uM of reparixin in CM from fibroblasts or macrophages with TCM of MDA-MB-231 cells using the Oris Cell migration kit. (TCM-F)CM and (TCM- MΦ)CM: conditioned media from fibroblasts or macrophages cultured with TCM (tumor conditioned media) of MDA-MB-231cells. (**C**) Migration assay of MDA-MB-231 cells (top chamber) treated 0.1 uM of reparixin with CM (bottom chamber) from fibroblasts or macrophages cultured with SFM containing with 2% serum or TCM of MDA-MB-231 cells in the RTCA system. The cell index was measured continuously at 48 h. (*P < 0.01, *n* = 3). (**D**) Tumor growth curves of MDA-MB-231 cells implanted mammary fat pad in athymic mice treated with 15 mg/kg reparixin *i.p*. daily for five weeks. (Mean ± SEM, **P < 0.005 and *n* = 10). (**E**) Athymic nude mice were pretreated with TCM (50 ul) of MDA-MB-231 cells for two weeks before inoculation with MDA-MB-231 cells and treatment of reparixin. After five weeks, the number of mice with thoracic metastasis was counted using the IVIS imager. The red box represents thoracic metastasis observed with the IVIS imager.

Next, we examined the viability of MDA-MB-231 cells co-cultured with fibroblasts or macrophages in the presence of reparixin, a CXCR1/2 inhibitor [28], and observed that reparixin decreased MDA-MB-231 cellular viability in a concentration-dependent manner (Figure 6A and Supplementary Figure 4A). The migration of MDA-MB-231 cells incubated with conditioned media of fibroblasts or macrophage induced by TCM of TNBC cells was inhibited by reparixin (Figure 6B–6C and Supplementary Figure 4B–4F) as well. In addition, the growth of MDA-MB-231 cell tumors treated with reparixin was significantly inhibited compared to control, and thoracic metastatic lesions were not detected in lungs and lymph nodes in these animals (Figure 6D–6E and Supplementary Figure 5A–5C). Furthermore, we observed that Ki-67 positive cells were significantly reduced in tumor treated with reparixin and the recruitment of fibroblasts and macrophages was decreased in the reparixin group compared to the control group using immunofluorescence and immunohistochemistry analysis of SMA, Iba-1, F4/80 and Ki-67. (Supplementary Figure 5C–5F). These results demonstrate that reparixin, which targets CXCR1/2, is a potential therapeutic drug to inhibit TNBC tumor growth and metastasis.

## DISCUSSION

Chemotherapy is the accepted systemic treatment for triple-negative breast cancer patients. Various therapies such as PARP inhibitors, anti-androgen therapy, PI3K inhibitors, MEK inhibitors, inhibitors of the cancer stem-cell population, EGFR inhibitors and HDAC inhibitors have been explored to cure TNBC patients because of the lack of specific TNBC therapies. In addition, immunotherapy utilizing immune-checkpoint inhibitors to target PD1/PDL-1 and CTLA-4 has recently shown potential benefits for TNBC patients [2], but a major molecular driver has not yet been identified. Here we have described findings that identify an important role of IL-8 in crosstalk between TNBC and stroma and propose IL-8 as a potential therapeutic target in TNBC. The elevated IL-8 cytokine as a tumor microenvironment factor has also been identified in the interstitial fluid collected in a tumor-implanted microchamber in TNBC xenografts, but not in ER+ xenografts, suggesting its specificity for TNBC [29].

The stromal components of the tumor microenvironment include cancer associated fibroblasts, blood endothelial cells, lymphatic endothelial cells, pericytes, and immune cells including tumor associated macrophages [30]. Cells secrete factors, the aggregate of which is called the secretome, which either regulate the cells secreting them in an autocrine fashion or regulate other cells in the vicinity or at a distance in a paracrine fashion. Secretomes play a major role in tumor growth and metastasis and critical targets that control these processes can be found in the secretomes [31]. The stromal cell secretome is altered by the tumor cell secretome and vice versa. In addition, it is likely that secretomes change in response to therapeutic drugs.

Cancer-associated fibroblasts produce extracellular matrix and reprogram immune surveillance in TME. The CAF is involved in tumor growth, metastasis and drug resistance [32, 33]. Tumor-associated macrophages are involved in tumor-promoting functions such as tumor cell proliferation, invasion, immunosuppression, and angiogenesis as well as anti-tumor functions such as direct tumor cell killing and NK- or T-cell mediated tumor cell killing [34].

In our previous study, we demonstrated a crosstalk between TNBC cells and lymphatic endothelial cells. We developed a tumor-conditioned regional lymph node (LN) model by injecting the tumor-conditioned media (TCM) subcutaneously into animals for 14 days to simulate the conditioning of tumor-draining LNs by primary tumor–secreted factors that are transported to the LNs through lymph node vessels [35, 36]. We found that the axillary and brachial LNs of TCM-treated animals were two to three times larger than those of SFM-treated animals [37]. Furthermore, we observed that pre-treatment of animals with TCM consistently accelerates spontaneous metastasis to the axillary and brachial lymph nodes (LN), and the lungs compared to the serum-free media treated group. The LN in TCM-treated animals showed enhanced angiogenesis and lymphangiogenesis [38]. The lymphatic endothelial cells are induced by tumor-conditioned media (TCM) of TNBC cells. Interleukin-6 (IL-6), a major cytokine in the TCM, interacts with the IL-6 receptor in LEC and activates the JAK2-STAT3 signaling pathway, which upregulates chemokine (C-C motif) ligand 5 (CCL5) and vascular endothelial growth factor (VEGF) in LEC. The secreted CCL5 binds to CCR5, the CCL5 receptor, which enhances TNBC metastatic properties and promotes metastasis to lymph nodes. VEGF helps angiogenesis in the lymph nodes, and it helps tumor cells extravasate into the lung. Based on these findings, we showed that inhibition of either CCR5 or VEGF blocks TNBC tumor metastasis to the lung [23]. These previous findings motivated our hypothesis that secreted factor(s) from crosstalk between TNBC and CAF or TAM could be therapeutic targets to treat TNBC. In line with this prediction, we found that co-cultures of three different TNBC cell lines with normal fibroblasts and M2 type macrophages promote cell proliferation. In addition, we observed that TNBC cell migration was enhanced by the conditioned media of fibroblasts or macrophages induced by TCM of TNBC cells (Figure 1). Our data implicated a crosstalk between TNBC cells and fibroblasts or macrophages and that this crosstalk promotes TNBC cell migration and proliferation.

The next interesting question was whether factors secreted by fibroblasts and macrophages regulate TNBC cell migration and proliferation. We screened the conditioned media of fibroblasts or macrophages induced by TCM of TNBC cells to identify a critical secreted factor using the human cytokine antibody array targeting 105 cytokines and discovered ten different putative cytokines that were upregulated as a result of exposure to TCM from TNBC cells. Through ELISA and QRT-PCR analysis, we identified IL-8 as the top secreted factor candidate that could increase TNBC cell migration and proliferation (Figure 2). We confirmed the key role of IL-8 by showing that when a neutralizing anti-IL-8 specific antibody was added to conditioned media of fibroblasts or macrophages induced by TCM of TNBC cells, these condition media were no longer able to increase TNBC cell proliferation and migration (Figure 3). We found that the proliferation rate of MDA-MB-231 cells was higher by about 1.5-fold when co-culture with fibroblast versus macrophages. Interestingly, we observed that the level of migration was increased about 2-fold in the conditioned media from macrophages induced by TCM compared to fibroblasts in Figures 1, 2 and 3. This difference demonstrated that IL-8 protein is a key factor to promote TNBC cell proliferation and migration via a crosstalk between TNBC and stroma, but IL-8 is not the only molecule. We discovered more candidates such as HGF, IL-6, CCL7, MIF, GDF-15, EMMPRIN, VEGF, CXCL5, and uPAR using the cytokine array. To find out how these factors contribute to TNBC tumor growth and metastasis will be worth considering in the future studies.

Probing this finding further, we investigated the IL-8 signaling pathway by performing experiments to perturb signaling from its receptors, CXCR1 and CXCR2. We next asked if it was possible to inhibit proliferation and/or migration of TNBC cells by reducing the levels of CXCR1 or CXCR2 in them. To answer this, we constructed CXCR2 knockout MDA-MB-231 cell line using the CRISPR-Cas9 system and showed that MDA-MB-231 cells with a CXCR2 knockout had significantly reduced rates of cell proliferation and migration compared to wild-type cells. In a xenograft mouse model, the growth of tumors from MDA-MB-231-CXCR2^−/−^ cells and metastasis from these tumors were dramatically decreased compared to tumors from wild type MDA-MB-231 cells. These observations imply that the axis of IL-8-CXCR2 signaling that occurs between TNBC cells and fibroblasts and macrophages plays an important role in TNBC tumor growth and metastasis (Figure 4).

The autocrine IL-8 loop activates AKT, MAPK and STAT3 signaling pathways in tumor cells, which in turn induce the upregulation of transcription factors such as Snail, Slug, and Twist. These transcription factors bind to the E-box in the IL-8 promoter to increase IL-8 expression. The paracrine IL-8 loop recruits TAM into the tumor microenvironment. The TAM induces secretion of cytokines, chemokines and growth factors [16]. These results led us to investigate how the IL-8 from TNBC cells activates stromal cells in a paracrine fashion, which, in turn, enhances secretion of IL-8 from the stromal cells. We found that IL-8 secretion from MDA-MB-231-CXCR2^−/−^cells is lower than from wild type MDA-MB-231 cells, suggesting the autocrine IL-8 loop is dependent on CXCR2. In addition, we observed the effect of paracrine IL-8 in which IL-8 secreted by MDA-MB-231-wild type cells activated the IL-8 signaling pathway in fibroblasts and macrophages. Consistent with these results, we also observed reduced activation of IL-8 signaling in fibroblasts and macrophages due to the reduction in autocrine IL-8 signaling in MDA-MB-231-CXCR2^−/−^ cells. These findings provide evidence that important, functional auto- and paracrine loops of IL-8 from the crosstalk between TNBC cells and stroma promote TNBC proliferation and migration (Figure 5).

In our search for clinically feasible approaches for inhibiting IL-8 signaling, we found reparixin, a known CXCR1/2 inhibitor, which has been reported to inhibit tumor growth and metastasis [39–41]. It has also been shown to affect inflammatory response, recovery of the traumatic lesion, blood pressure and brain damage [42–45]. We found that reparixin indeed inhibited MDA-MB-231 tumor growth and metastasis suggesting that it could be a potential therapeutic drug to treat TNBC (Figure 6).

In summary, our current model is that TNBC cells secrete IL-8 which interacts with the CXCR1/2 receptor on tumor associated fibroblasts and macrophages in the tumor microenvironment. Activated CXCR1/2 receptors induce phosphorylation of STAT3, which results in upregulated IL-8 expression and secretion from these stromal cells. The IL-8 secreted by these tumor associated fibroblasts and macrophages interacts with the CXCR1/2 receptor on TNBC cells enhancing TNBC tumor growth and metastatic extravasation and colonization. Taken together, these findings contribute significantly to our understanding of the role of IL-8 signaling as a critical event in TNBC tumor growth and metastasis via crosstalk with stromal components. Further, these studies suggest that IL-8 acts as a key regulator orchestrating TNBC metastatic breast cancer. Therefore, we have provided evidence that supports the hypothesis that functional antagonism of the IL-8 signaling pathway has the potential to circumvent TNBC breast cancer growth and metastasis.

## MATERIALS AND METHODS

### Cell Lines

MDA-MB-231-luc-D3H2LN cells were purchased from Caliper and propagated in RPMI-1640 medium supplemented containing with 10% FBS and 1% penicillin/streptomycin (Sigma, St. Louis, MO). These cells are labeled as MDA-MB-231 throughout the text for brevity. SUM149 and SUM159 breast cancer cells were gifts from Dr. Zaver Bhujwalla (Radiology and Oncology, Johns Hopkins Medical Institutes). Cells were cultured in F-12 media supplemented containing with 5% FBS, 1 ng/ml hydrocortisone, 5 μg/ml insulin (Sigma, St. Louis, MO), and 0.1mM HEPES (ThermoFisher Scientific, Waltham, MA). Normal human lung fibroblasts (NHLF) were purchased from Lonza and grown in DMEM medium supplemented containing with 10% FBS and 1% penicillin/streptomycin (Sigma, St. Louis, MO). Peripheral Blood Mononuclear Cells (PBMCs) were purchased from Zenbio and Monocytes were differentiated into M2 type macrophages by culturing for 6 days in RPMI medium supplemented containing with10% FBS, 100 ng/ml of M-CSF. For generating the CXCR2 knockout MDA-MB-231 cell line using the CRISPR-Cas9 system, sgRNA/Cas9 all-in-one expression clone targeting CXCR2 (GeneCopoeia, Rockville, MD) was stably transfected into MDA-MB-231 cells using Lipofectamine 3000 (ThermoFisher Scientific, Waltham, MA). Cells were maintained under standard conditions of 37°C and 5% CO2. Cells were cultured for a maximum of 4 weeks before thawing fresh, early passage cells and confirmed to be Mycoplasma negative (Hoechst stain).

### Conditioned media

When MDA-MB-231, SUM149, and SUM159 cells were confluent in T175 tissue culture flasks, the normal cancer cell growth media was replaced with 8 ml serum-free media (SFM) containing with 2% serum. After 24 h incubation in a tissue culture incubator, the supernatant was centrifuged and filtered through 0.2 μm syringe filters (Corning, Corning, NY). The resulting tumor-conditioned media (TCM) was stored in aliquots at −80°C. When fibroblasts and macrophages reached 30–40% confluence in T75 tissue culture flasks, the growth medium (GM) was replaced with 30% TCM in GM (TCM: GM = 3:7) to allow the TCM to induce the fibroblast cells and macrophage cells. For the education, the cells were allowed to grow in the media for 3–4 days then the media was replaced with 3 ml SFM containing with 2% FBS. After 48 h, the supernatant was centrifuged and filtered. The resulting tumor-induced fibroblast and macrophage (MDA-MB-231- fibroblast or macrophage) conditioned media was stored in aliquots at −80°C to avoid multiple freeze-thaws.

### Immunoblotting

For reverse western blot, a human cytokine antibody array kits (R&D Systems) were used, according to the manufacturer's instructions. The western blotting analysis was performed with anti-STAT3, anti-Phospho STAT3, anti-CXCR2 (Cell Signaling, Danvers, MA), and anti-Actin antibody (Sigma, St. Louis, MO) as previously described [46].

### Cell migration and proliferation assays

Cancer cell migration was assessed by using the Oris^TM^ Cell migration kit (Platypus Technology, Madison, WI), as previously described [38]. MDA-MB-231- fibroblast or macrophage conditioned media (100 μl) with or without 0.1 uM reparixin (MCE, Monmouth Junction, NJ) and anti-IL-8 antibodies (R&D Systems, 30 μg/ml) was added once the cancer cells had attached. Migration and proliferation assays using CIM-plates (Roche, Indianapolis, IN) and the RTCA system (ACEA Bioscience, San Diego, CA) were performed as previously described [47].

### Mouse xenograft studies

Animal protocols described in this study were approved by the Institutional Care and Use Committee at the Johns Hopkins Medical Institutions. Before tumor inoculation, twenty athymic nude mice (female, 5–6 weeks, 18–20 g) were pre-treated by injecting 50 μl tumor-conditioned media (TCM) subcutaneously for two weeks daily as described previously [23]. After two weeks of induction, we then divided 10 mice into each two groups as follows: 1) wild-type and 2) CXCR2 knockout, or 1) control and 2) reparixin. 2 × 10^6^ MDA-MB-231 cells were injected into the upper inguinal mammary fat pad of the animals with 100 ul of 1:1 PBS/Matrigel (BD Biosciences, San Jose, CA). The tumor size was measured by using a caliper, and the volume was calculated, using the formula: V = 0.52 × (length) × (width)^2^. Animals were imaged every week to track thoracic metastases, using the IVIS Xenogen 200 optical imager (Xenogen, Alameda, CA) after i.p. Injection of D-luciferin (Caliper, Waltham, MA, 150 mg/kg). After five weeks, organs were harvested and bathed in D-luciferin solution for 5–10 min and placed in the IVIS imager to detect metastases *ex vivo*. Luciferase-mediated photon flux was quantified by using Living Image^®^ 3D Analysis (Xenogen, Alameda, CA). reparixin (15 mg/kg, MCE) was administered intraperitoneally daily for five weeks.

### Immunofluorescence and immunohistochemistry

Immunofluorescence and immunohistochemical analysis of Iba-1 and αSMA were performed using monoclonal antibodies against Iba-1 (Wako) and αSMA-Cy3 (Sigma, St. Louis, MO). For immunofluorescence, after blocking with 5% normal goat or normal chicken serum (Jackson Immunoresearch, West Grove, PA) in PBST (0.3% Triton) for 1 h at room temperature (RT), the sections were treated with Iba-1 and αSMA primary antibodies overnight at 4°C. After 3 rinses with PBST, sections were incubated for 1 hour at RT with FITC-conjugated goat anti-rabbit secondary antibodies (1:500). After three rinses with PBST, the samples were counterstained with DAPI (1:10,000, Roche) (5 min at RT). The samples were washed with PBST once and mounted with the ProLong Gold anti-fade reagent (Invitrogen) in the dark. Fluorescent signals were visualized and digital images were obtained using the Zeiss LSM-700 confocal microscope (Carl Zeiss, Oberkochen, Germany). For IHC, after blocking with 5% goat serum in PBST for 1 hour at room temperature, the sections were treated with the Iba-1 and αSMA antibodies overnight at 4°C, then the peroxidase-conjugated streptavidin complex method was performed, followed by the 3, 3′ diaminobenzidine (DAB) procedure according to manufacturer's protocols (VECTASTAIN Elite ABC Kit, Vector Lab, Burlingame, CA).

### Statistical analysis

Each experiment was repeated at least three times. The results of three independent experiments performed in triplicate were shown as mean ± SD or SEM compared with control. All statistical analyses were performed using a two-sided unpaired *t*-test using GraphPad Prism 6 (Graphpad, La Jolla CA). A *p*-value of 0.05 or less was considered significant.

## SUPPLEMENTARY FIGURES


