# Supplementary material for: Crosstalk between stromal components and tumor cells of TNBC via secreted factors enhances tumor growth and metastasis
Source: Oncotarget. 2017 Jul 21;8(36):60210–22. doi: 10.18632/oncotarget.19417 (PMC5601133; doi:10.18632/oncotarget.19417)
Supplement: Supplementary file 1 [file oncotarget-08-60210-s001.pdf]

Crosstalk between stromal components and tumor cells of TNBC via secreted factors enhances tumor growth and metastasis

SUPPLEMENTARY MATERIALS

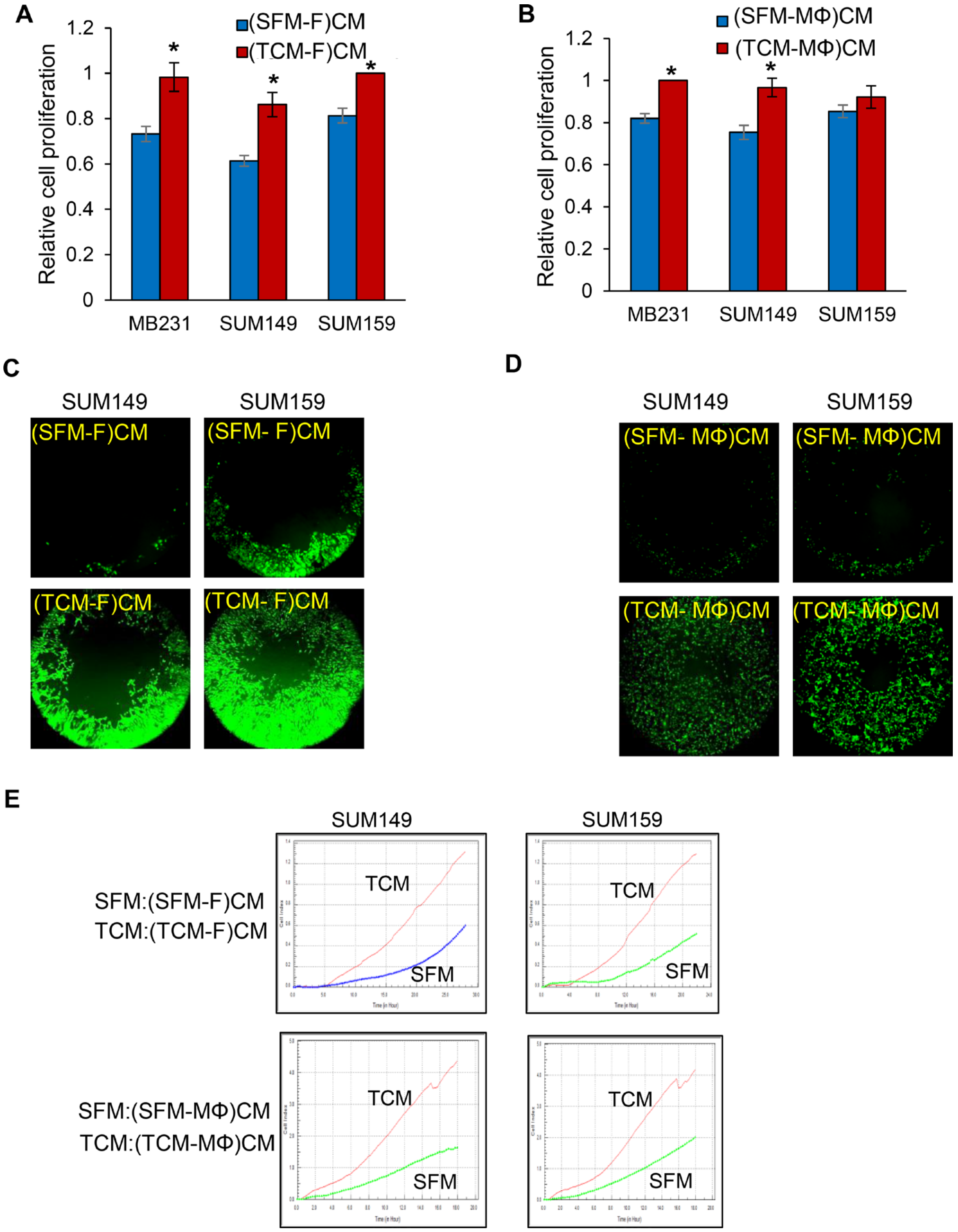

Supplementary Figure 1: (**A**) MTT assay on three different TNBC cells including MDA-MB-231, SUM149 and SUM159 cells with CM from fibroblasts or (**B**) macrophages cultured with SFM or TCM of each TNBC cell. (T: TNBC cells, **P* < 0.05 and *n* = 3). (**C**) Migration assay on SUM149 and SUM159 cells with CM from fibroblasts or (**D**) macrophages cultured with SFM or TCM of each TNBC cell. Migration was measured at 48 h using the Oris Cell migration kit. (**E**) Migration assay on SUM149 and SUM159 cells (top chamber) with CM (bottom chamber) from fibroblasts or macrophages cultured with SFM or TCM of each TNBC cell in the RTCA system. The cell index was measured continuously for 48 h.

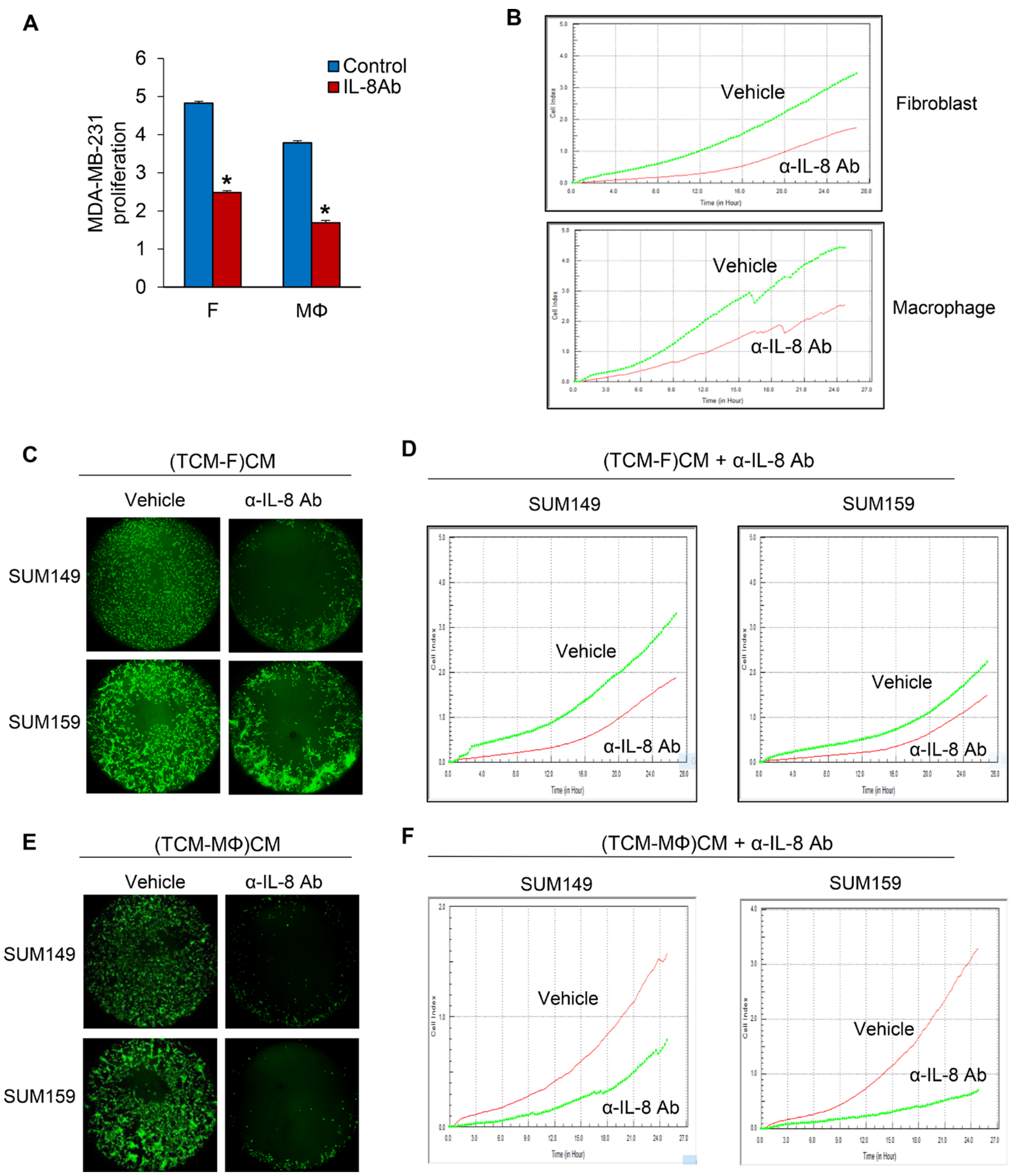

Supplementary Figure 2: (**A**) Proliferation assays on MDA-MB-231 cells (bottom chambers) co-cultured with fibroblasts and macrophages treated with anti-IL-8 antibody (R&D Systems) on the top chamber in the RTCA system (**P* < 0.001, *n* = 3). (**B**) Migration assay on MDA-MB-231 cells treated with anti-IL-8 antibody in CM from fibroblasts or macrophages cultured with SFM or TCM of MDA-MB-231 cells. The cell index was measured continuously for 48 h. (**C**) Migration assay on SUM149 and SUM159 cells treated with anti-IL8 antibody in CM from fibroblasts cultured with SFM or TCM of each TNBC cell using the Oris cell migration kit and (**D**) Oris assay in C was done on the RTCA system for 48 hours. (**E**) and (**F**) The same assays as C and D were performed with macrophages.

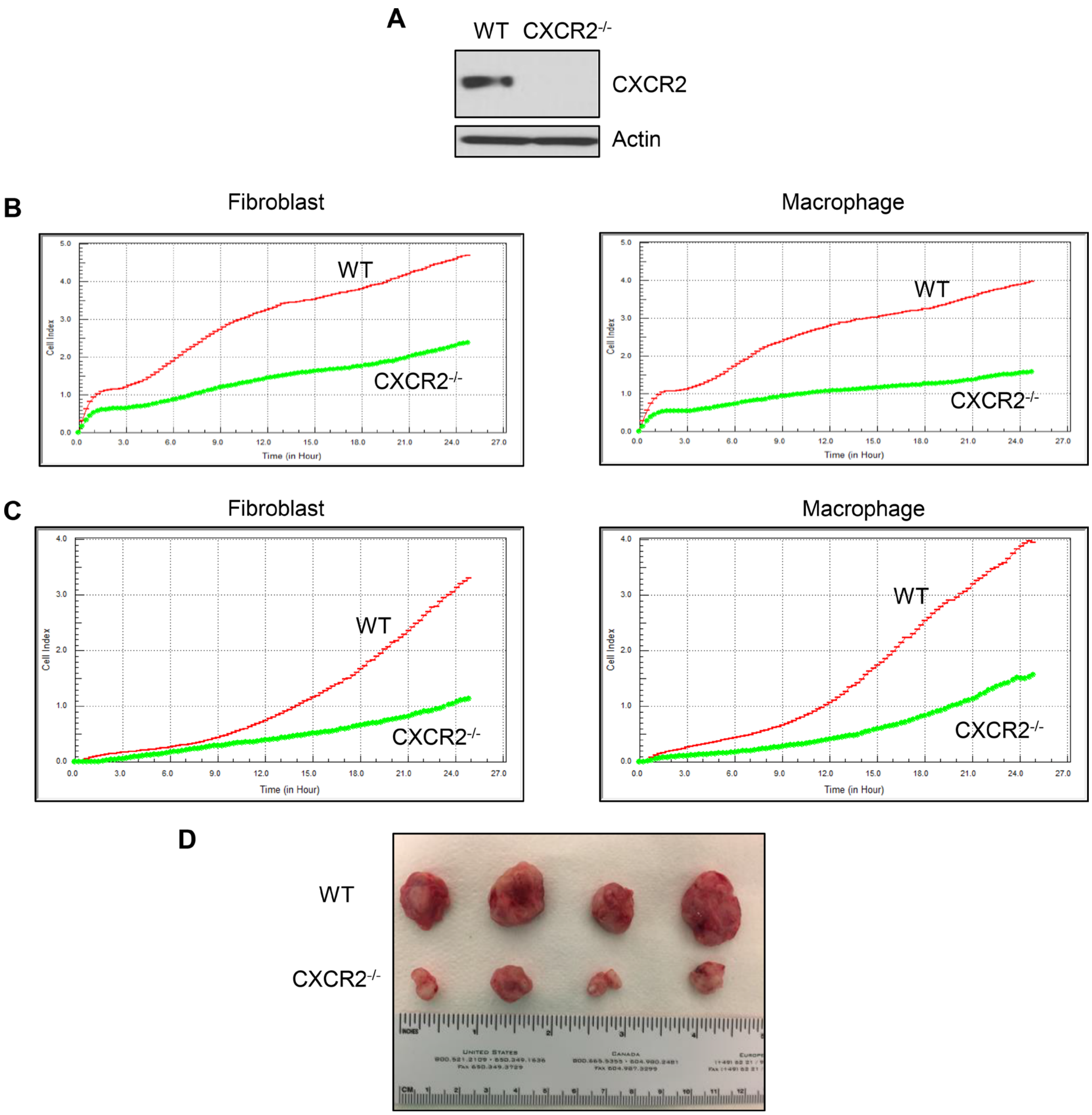

A
WT
CXCR2-/-
CXCR2
Actin
B
Fibroblast
Macrophage
WT
CXCR2-/-
Cell Index
Time (in Hour)
C
Fibroblast
Macrophage
WT
CXCR2-/-
Cell Index
Time (in Hour)
D
WT
CXCR2-/-

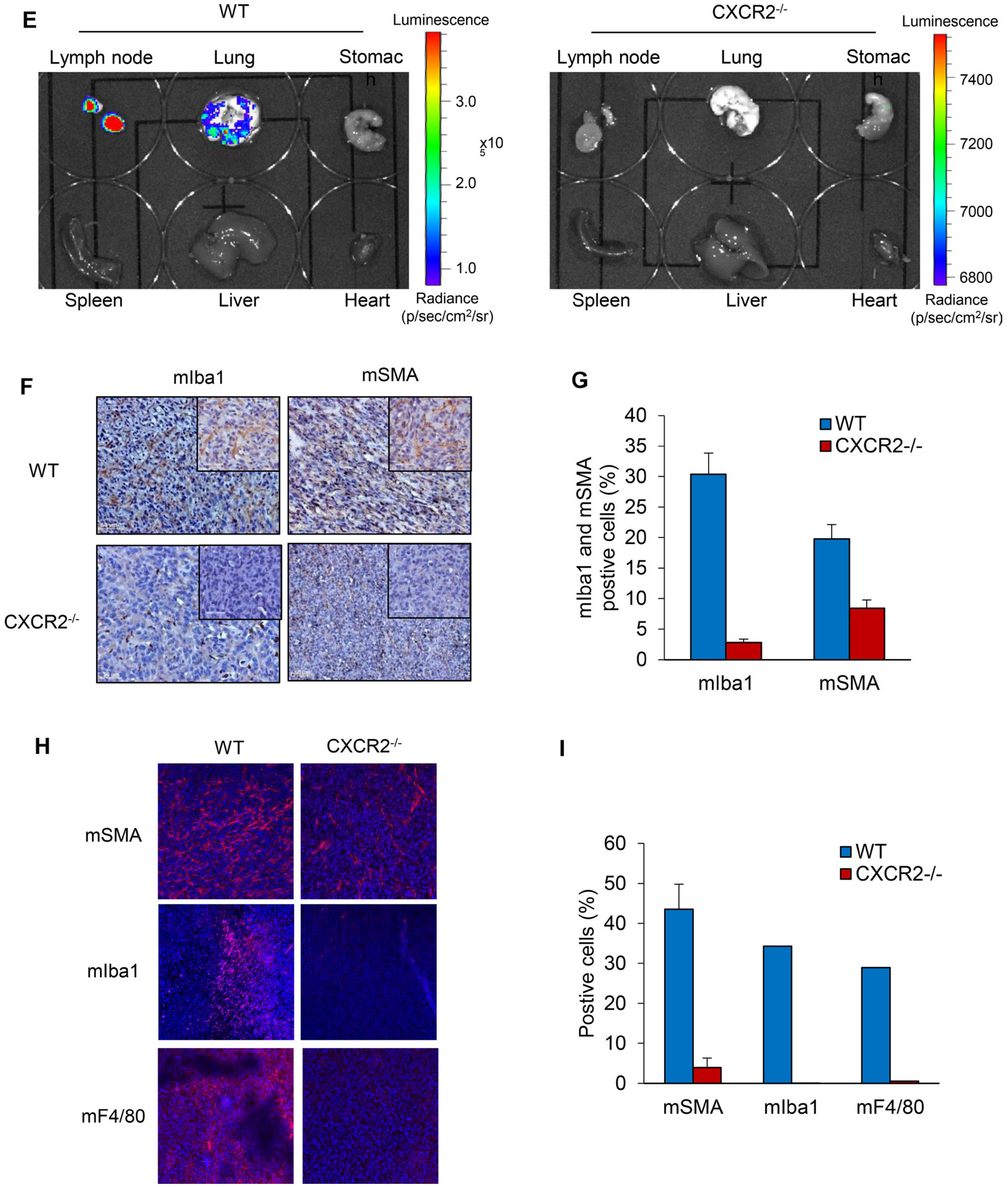
E
WT
Luminescence
Lymph node
Lung
Stomac
h
Spleen
Liver
Heart
3.0
2.0
1.0
x10
5
Radiance
(p/sec/cm2/sr)
CXCR2-/-
Luminescence
Lymph node
Lung
Stomac
h
Spleen
Liver
Heart
7400
7200
7000
6800
Radiance
(p/sec/cm2/sr)
F
mIba1
mSMA
WT
CXCR2-/-
G
WT
CXCR2-/-
mIba1 and mSMA postive cells (%)
40
35
30
25
20
15
10
5
0
mIba1
mSMA
H
WT
CXCR2-/-
mSMA
mIba1
mF4/80
I
WT
CXCR2-/-
Postive cells (%)
60
50
40
30
20
10
0
mSMA
mIba1
mF4/80

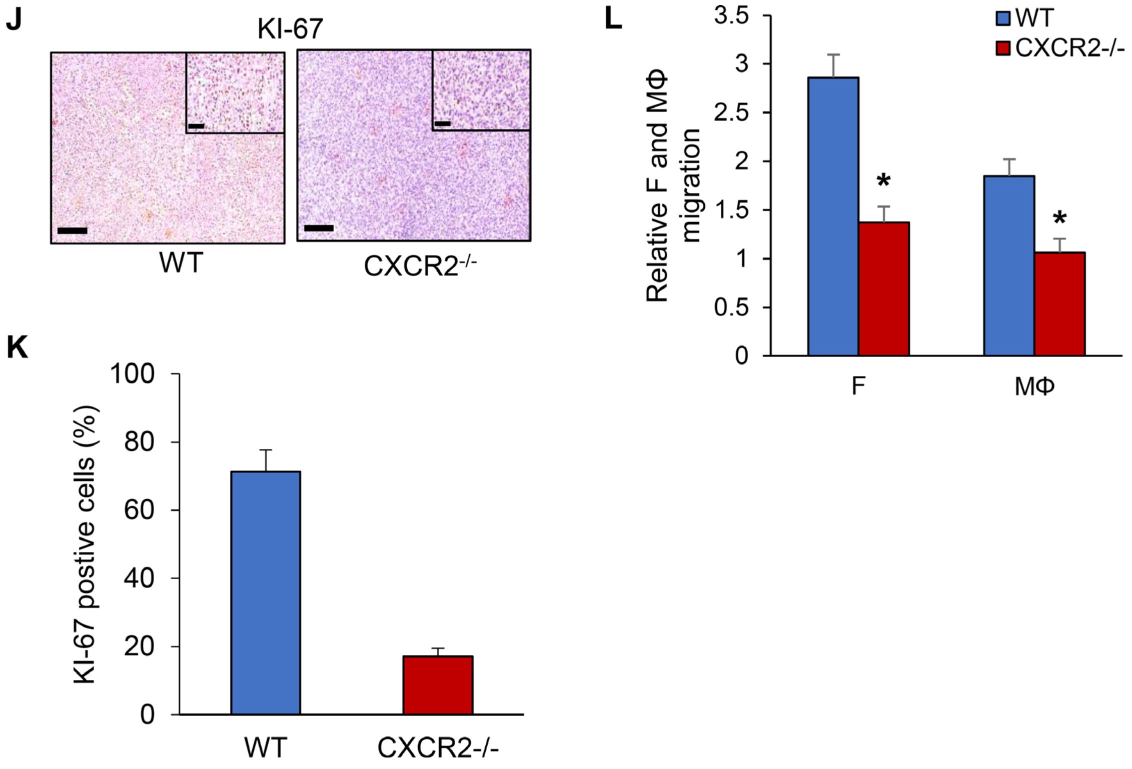

Supplementary Figure 3: (**A**) Immunoblot analysis of CXCR2 in MDA-MB-231-WT and MDA-MB-231-CXCR2-/- cells using anti-CXCR2 antibody. (**B**) Proliferation assays on MDA-MB-231-WT and MDA-MB-231-CXCR2-/- cells (bottom chambers) co-cultured with fibroblasts or macrophages on the top chamber in the RTCA system. The cell index was measured continuously for 48 h. (**C**) Migration assay on MDA-MB-231-WT and MDA-MB-231-CXCR2-/- cells in CM from fibroblasts or macrophages cultured with SFM or TCM of MDA-MB-231-WT cells. The cell index was measured continuously for 48 h. (**D**) Size of MDA-MB-231-WT and MDA-MB-231-CXCR2-/- cells xenografts in athymic nude mice after 5 weeks. (**E**) Representative images of luciferase-mediated photon flux from lung, lymph node, stomach, spleen, liver and heart. (**F**) Representative IHC image of Iba-1 and αSMA in tumors, and (**G**) results from F were examined and quantified. (**H**) Representative IF images of mouse Iba-1, αSMA and F4/80 in tumors, and (**I**) results from H were examined and quantified. (**J**) Representative IHC image of Ki-67 in tumors, and (**K**) results from J were examined and quantified. (**L**) Migration assay on fibroblasts or macrophages (top chamber) with TCM (bottom chamber) from WT and CXCR2-/- MDA-MB-231 cells using the RTCA system. (*$P < 0.01$, $n = 3$).

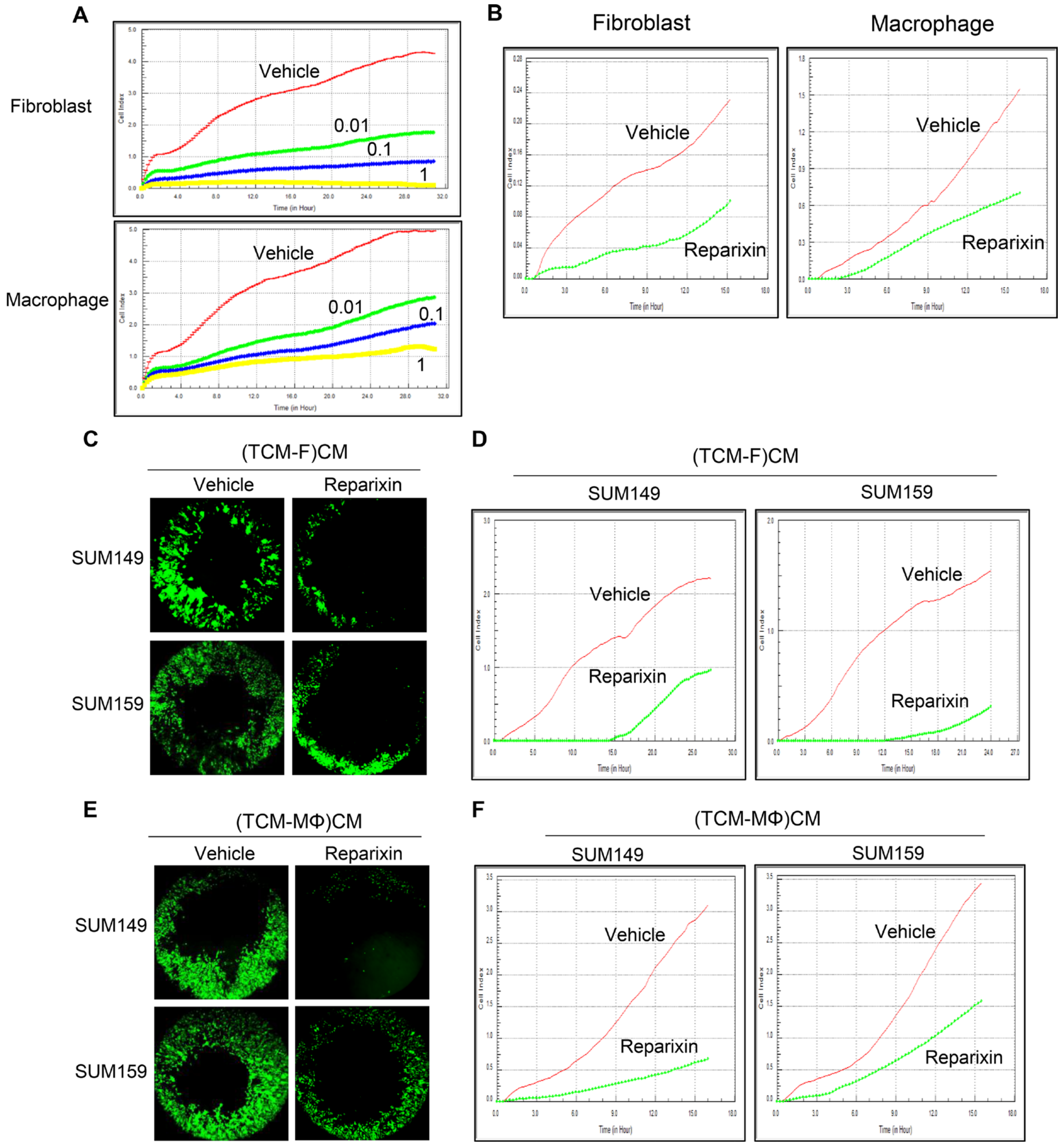

Supplementary Figure 4: (**A**) Proliferation assays on MDA-MB-231 cells (bottom chambers) co-cultured with fibroblasts and macrophages treated with 0.01 to 1 uM of reparixin on the top chamber in the RTCA system. The cell index was measured continuously for 48 h. (**B**) Migration assay on MDA-MB-231 cells treated with reparixin in CM from fibroblasts or macrophages cultured with SFM or TCM of MDA-MB-231 cells. The cell index was measured continuously for 48 h. (**C**) Migration assay on SUM149 and SUM159 cells treated with reparixin in CM from fibroblasts cultured with SFM or TCM of each TNBC cell using the Oris cell migration kit and (**D**) RTCA system for 48 hours. (**E**) and (**F**) The same assay was performed with macrophages as C and D.

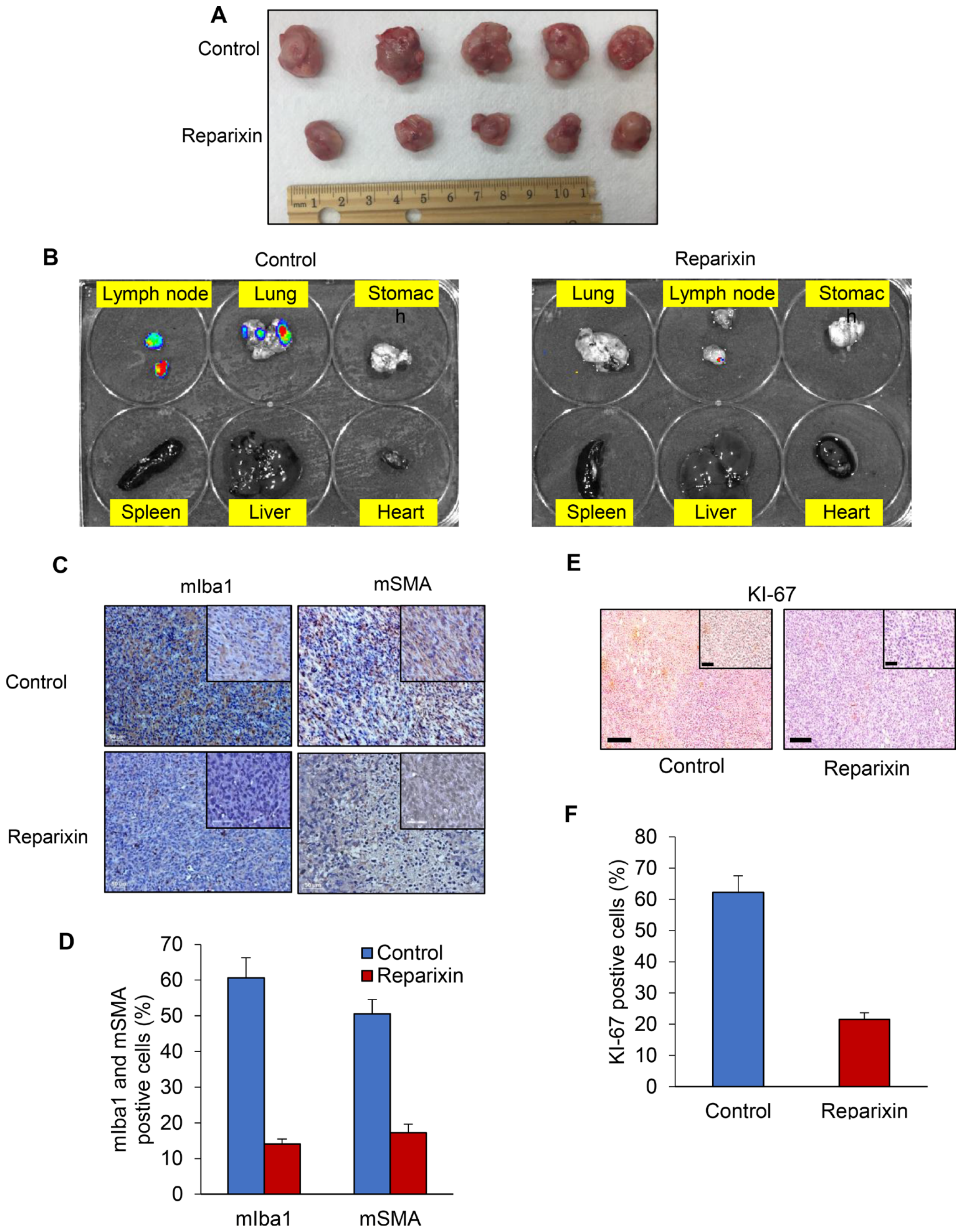

Supplementary Figure 5: (**A**) Size of MDA-MB-231 cells xenografts in athymic nude mice after 5 weeks of treatment with control and reparixin. (**B**) Representative images of luciferase-mediated photon flux from lung, lymph node, stomach, spleen, liver and heart. (**C–F**) Representative IHC images of mouse Iba-1, αSMA, and KI-67. Results were examined and quantified.

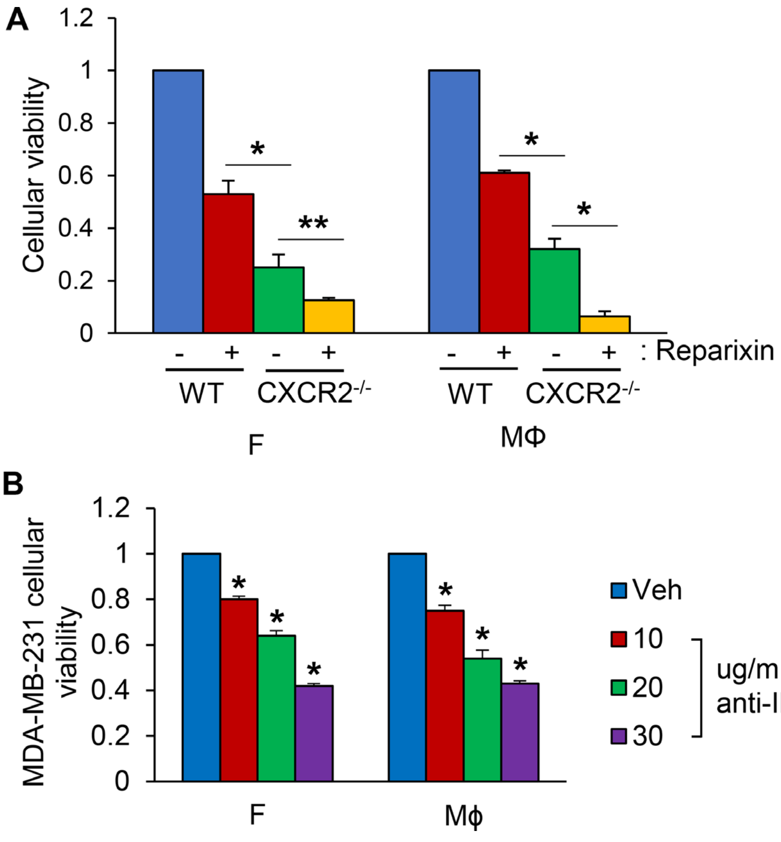

Supplementary Figure 6: (**A**) Cellular viability assays on MDA-MB-231-wild type and MDA-MB-231-CXCR2-/- cells (bottom chambers) with fibroblasts or macrophages treated with 0.01 uM of reparixin on the top chamber in the RTCA system. (**B**) Cellular viability assays on MDA-MB-231 cells (bottom chambers) co-cultured with fibroblasts and macrophages treated with 10 to 30 ug/ml of anti-IL-8 antibody on the top chamber in the RTCA system (**P* < 0.005, ***P* < 0.05, *n* = 3).

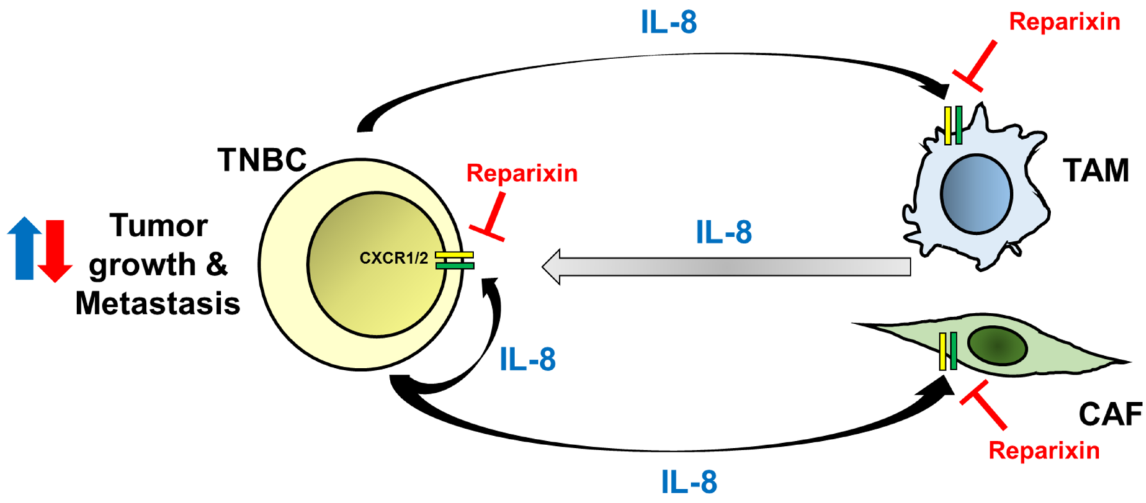

Supplementary Figure 7: A model of the crosstalk between TNBC and cancer associated fibroblasts (CAF) or tumor associated macrophages (TAM). IL-8 secretion from TNBC recruits CAF and TAM, and enhances their IL-8 protein expression and secretion. IL-8 secreted from CAF and TAM triggers activation of the IL-8-CXCR1/2 signaling axis in TNBC cells and promotes IL-8 expression as a consequence. This positive feedback between IL-8 protein and CXCR1/2 enhances TNBC tumor growth and metastasis. The IL-8-CXCR1/2 axis could thus be a potential therapeutic target. In support of this model we have shown that reparixin, a CXCR1/2 inhibitor, blocks TNBC tumor growth and metastasis.
